# Pattern and trend of five major musculoskeletal disorders in China from 1990 to 2017: findings from the Global Burden of Disease Study 2017

**DOI:** 10.1186/s12916-021-01905-w

**Published:** 2021-02-04

**Authors:** Dongze Wu, Priscilla Wong, Cui Guo, Lai-Shan Tam, Jieruo Gu

**Affiliations:** 1 Department of Rheumatology, The Third Affiliated Hospital of Sun Yat-sen University, No. 600 Tianhe Road, Tianhe District, Guangzhou, 510000 Guangdong China; 2 Department of Medicine & Therapeutics, The Prince of Wales Hospital, The Chinese University of Hong Kong, 9F, LCW Clinical Sciences Building, Hong Kong SAR, China; 3 Jockey Club School of Public Health and Primary Care, The Chinese University of Hong Kong, Hong Kong SAR, China

**Keywords:** Temporal trend, Disease burden, Rheumatoid arthritis, Osteoarthritis, Low back pain, Gout

## Abstract

**Background:**

With increasing life expectancy in China, no large population-based studies have been done on the trend for musculoskeletal disorders in China. We have investigated the pattern and trend of five major musculoskeletal disorders in China from the Global Burden of Disease Study 2017 and its association with sociodemographic index (SDI).

**Methods:**

The main outcome measures were incidence, prevalence, and disability-adjusted life years (DALYs) for rheumatoid arthritis, osteoarthritis, low back pain, neck pain, and gout. Average annual percent change (AAPC) and annual percent change (APC) between 1990 and 2017 were analyzed with Joinpoint regression.

**Results:**

The age-standardized rate of incidence, prevalence, and DALYs for the five major musculoskeletal disorders increased with age. For SDI, the age-standardized rate of DALYs was zigzagged increasing for rheumatoid arthritis and curvilinear increasing for gout, curvilinear decreasing for low back pain, and reaching to the highest point for osteoarthritis and neck pain with an SDI value of 0.61. The AAPC in age-standardized rate of DALYs indicated an increasing trend for rheumatoid arthritis (0.20, 95% CI 0.07, 0.34), osteoarthritis (0.26, 95% CI 0.20, 0.31), neck pain (0.09, 95% CI 0.07, 0.12), and gout (0.25, 95% CI 0.23, 0.27), but a decreasing trend for low back pain (− 0.96, 95% CI − 0.98, − 0.93). The AAPC of risk factors indicated a decreasing trend in smoking (− 0.14, 95% CI − 0.24, − 0.04) for rheumatoid arthritis, smoking (− 0.22, 95% CI − 0.24, − 0.19) and occupational ergonomic factors (− 1.25, 95% CI − 1.29, − 1.21) for low back pain, and impaired kidney function (− 0.95, 95% CI − 1.00, − 0.90) for gout, but an increasing trend in high body-mass index for osteoarthritis (3.10, 95% CI 3.03, 3.17), low back pain (3.07, 95% CI 2.99, 3.14), and gout (3.12, 95% CI 3.04, 3.20). Comparing the burden of five musculoskeletal diseases in China with the 19 countries of G20, China ranked first to second in the number of DALYs, and 12th to 16th in age-standardized rate of DALYs.

**Conclusion:**

There are remarkably complex temporal patterns in disease burden and risk factors for five major musculoskeletal disorders across past three decades. Population-wide initiatives targeting high body-mass index may mitigate the burden of musculoskeletal disorders.

**Supplementary Information:**

The online version contains supplementary material available at 10.1186/s12916-021-01905-w.

## Background

Population aging has become an important aspect of the transition underway in China [1], which has been driven by a rapid decline in fertility rate from the 1970s and declining age-specific mortality rates [2]. In China, between 1950 and 2017, life expectancy increased from 47.5 to 74.5 years for men and from 51.3 to 79.9 years for women, despite the massive setback around the famine in 1960 [3]. With increasing life expectancy globally, more effort is needed to maximize healthy life expectancy, namely, the additional years of life gained are spent in good health [4]. Musculoskeletal diseases pose major threats to healthy aging by limiting physical and mental capacities and functional ability, which incurred considerable economic and medical burdens to individuals, families, and governments [5]. The Global Burden of Disease Study shows that the prevalence and burden from musculoskeletal disorders are exceptionally high throughout the world [3, 4, 6]. In 2017, one of the three leading causes of years lived with disability in China was musculoskeletal disorders [1]. Five major musculoskeletal conditions, including rheumatoid arthritis, osteoarthritis, low back pain, neck pain, and gout, contribute to the largest proportion of musculoskeletal burden.

The sustainable development goals (SDGs) and the Decade of Healthy Ageing 2020–2030 [7] offer a timely and favorable opportunity for increasing global awareness on the importance of maintaining musculoskeletal health. The Healthy China 2030 plan declared to make public health a precondition for all future economic and social development [8]. All these blueprints aimed to improve longevity and healthy life expectancy and to control major risk factors for chronic diseases [8]. Close examination of musculoskeletal burden at the national level will be crucial to developing evidence-based policies. Although exposures to behavioral, environmental, and occupational risks declined, it was somewhat offset by increases in exposure to metabolic risks, population growth, and population aging [9]. Thus, the drivers in risk-attributable burden of five major musculoskeletal diseases are crucial for risk prevention and attention to upstream determinants of health.

The Global Burden of Diseases, Injuries, and Risk Factors Study 2017 (GBD 2017) comprehensively and systematically quantifies the comparative magnitude of health loss due to diseases and injuries and provides a standardized approach for estimating musculoskeletal burden in China [3, 4, 6, 9]. The current study aims to (1) characterize the cross-sectional pattern of five major musculoskeletal disorders, (2) determine the temporal trend in the five major musculoskeletal disorders, and (3) evaluate the association between societal development and disease burden in China.

## Methods

### Data sources

The general methodological approaches used in GBD 2017 and the specific methodology used for the five major musculoskeletal disorders (rheumatoid arthritis, osteoarthritis, low back pain, neck pain, and gout) have been described elsewhere [3, 4, 6, 9]. Data from the GBD 2017 from 1990 to 2017 were used to analyze the burden of five major musculoskeletal disorders in China. GBD 2017 estimated incidence, prevalence, and disability-adjusted life years (DALYs) of various conditions by age, sex, cause, and year using a wide range of standardized analytical procedures, including data screening, data adjustment, and DisMod-MR 2.1 estimation [3, 4, 6, 9].

Morbidity (years lived with disability, YLDs) and mortality (death) estimates were added to yield DALYs, which are reported as numbers, and the DALY rate per 100,000 persons [3, 6]. The DALYs for osteoarthritis, low back pain, neck pain, and gout did not consider mortality as there was no evidence for cause-specific mortality associated with them. Disability weighting was derived from general population-based surveys [10], and all age-standardized results were calculated based on the GBD reference [4].

### Case definition

International Classification of Diseases, Ninth Revision (ICD-9) and ICD-10 codes were used to define rheumatoid arthritis (ICD9: 714.0–714.9; ICD10: M05, M06, M08), osteoarthritis (ICD9: 715; ICD10: M16, M17), low back pain (ICD9: 724; ICD10: M54.3, M54.4, M54.5), neck pain (ICD9: 723.1; ICD10: M54.2), and gout (ICD9: 274; ICD10: M10), respectively.

### The sociodemographic index

The sociodemographic index (SDI) is a summary measure of societal development, which was constructed using the Human Development Index methodology [6]. The national SDIs for China between 1990 and 2017 ranged from 0.46 to 0.71 [1].

### Risk factors and population attributable fraction

GBD 2017 identified risk factors for the five major musculoskeletal disorders, namely smoking for rheumatoid arthritis; high body-mass index (BMI) for osteoarthritis; smoking, high BMI, and occupational ergonomic factors for low back pain; and high BMI and impaired kidney function for gout. High BMI was defined as BMI ≥ 25 kg/m^2^ for adults (aged 20+ years) and using thresholds from the International Obesity Task Force standards for children (aged < 20 years) [9]. The population attributable fractions (PAFs) quantifies the contribution of a risk factor to a disease or a death and represents the proportion of outcomes that would be reduced in any given year if exposure to a risk factor in the past had been reduced to the counterfactual level of theoretical minimum risk exposure [9, 11]. For a given risk-outcome pair, the attributable DALYs was estimated as total DALYs for the outcome multiplied by the PAF for the risk-outcome pair for each age, sex, location, and year [9].

### Statistical analysis

The number and rate of incidence, prevalence, and DALYs with 95% uncertainty intervals (UIs) of the 5 major musculoskeletal diseases were reported according to age and gender. Age-standardized rate of incidence, prevalence, and DALYs were plotted against SDI between 1990 and 2017. Simple correlation coefficients were used to examine associations. Temporal trend changes were determined using a Joinpoint regression model. Average annual percent change (AAPC) was calculated for the entire period analyzed, and annual percent change (APC) was calculated for each segmented line regression with maximal number of Joinpoint of three [12]. The temporal trends were defined according to the statistical significance of the AAPC compared to zero. A large increasing trend was defined as AAPC or APC ≥ 1 and a large decreasing trend was defined as AAPC or APC ≤ − 1, vice versa. Any AAPC with a 95% CI overlapping with zero was considered stable. All statistical analyses were performed with Joinpoint Regression Program (version 4.8.0.1, Statistical Methodology and Applications Branch, Surveillance Research Program, National Cancer Institute) with *P* values less than 0.05 considered statistically significant.

## Results

### Cross-sectional analysis

From 1990 to 2017, the five most common musculoskeletal disorders in China were rheumatoid arthritis, osteoarthritis, low back pain, neck pain, and gout. The most burdensome in terms of DALYs were neck pain (8,758,013 [95% UI 6,066,846–12,292,429]), low back pain (7,184,540 [95% UI 5,081,105–9,735,270]), osteoarthritis (1,971,782 [95% UI 983,944–3,935,656]), rheumatoid arthritis (678,767 [95%UI 509,013–860,809]), and gout (264,729 [95% UI 178,292–363,196]) (Additional file: sTable 1). The age-standardized rate of DALYs per 100,000 persons due to neck pain, low back pain, osteoarthritis, rheumatoid arthritis, and gout were 466 (95% UI 323–650), 411 (95% UI 294–554), 99 (95% UI 49–198), 35 (95% UI 27–45), and 14 (95% UI 9–19), respectively (Additional file: sTable 1). The incidence and the prevalence for rheumatoid arthritis, osteoarthritis, low back pain, neck pain, and gout in terms of absolute number, and age-standardized rate are showed in Additional file: sTable 1.

Smoking contributed 8.0% (95% UI 2.7, 13.2), 9.4% (95% UI 3.1, 15), and 12.2% (95% UI 4.0, 20.1) towards the age-standardized rate of YLDs, DALYs, and death caused by rheumatoid arthritis. High BMI accounted for 16.2% (95% UI 7.0, 28.4) of age-standardized rate of DALYs due to osteoarthritis. Smoking, high BMI, and occupational ergonomic factors account for 14.6% (95% UI 10.9, 18.4), 4.5% (95% UI 2.1, 7.6), and 26.1% (95% UI 23.3, 29.2) of the age-standardized rate of DALYs due to low back pain. High BMI and impaired kidney function accounted for 24.0% (95% UI 11.1, 41.1) and 7.7% (95% UI 6.6, 8.9) of the age-standardized rate of DALYs due to gout (Additional file: sTable 2).

**Fig. 1:**
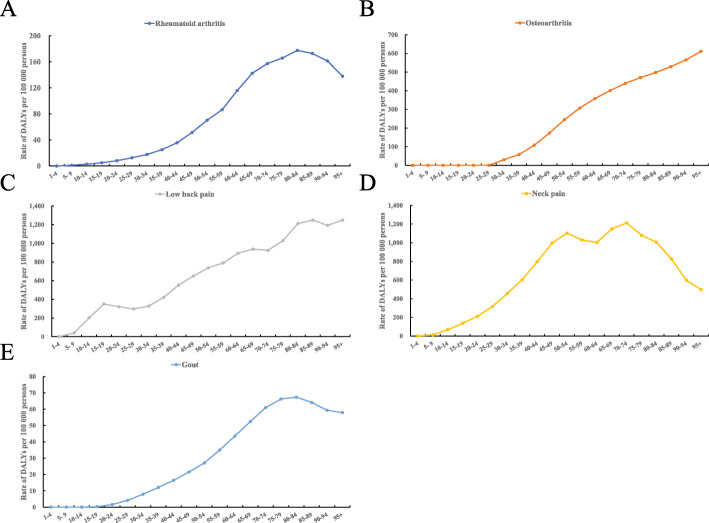
Rate of DALYs for 5 major musculoskeletal disorders at all ages. The age-stratified breakdown in rate of the DALYs for rheumatoid arthritis (**a**), osteoarthritis (**b**), low back pain (**c**), neck pain (**d**), and gout (**e**). Twenty age groups from 1 to 100 years old. All data are expressed as the rate of DALYs per 100,000 persons

With increasing age, the DALYs due to rheumatoid arthritis (Fig. 1a), neck pain (Fig. 1d), and gout (Fig. 1e) increased to a peak point and turn to decrease, due to osteoarthritis (Fig. 1b), and low back pain (Fig. 1c) increased steadily. Similar results were also observed for the incidence and prevalence for rheumatoid arthritis (Additional file: sFigure 1-A, B), low back pain (Additional file: sFigure 1-E, F), neck pain (Additional file: sFigure 1-G, H), and gout (Additional file: sFigure 1-I, J). In contrast, the incidence due to osteoarthritis sharply increased from age 25 to 55 and turned to slowly decrease from age 55 to 80 (Additional file: sFigure 1-C), while the prevalence due to osteoarthritis steadily increased from age 25 to 100 (Additional file: sFigure 1-D).

The 5 major musculoskeletal burden remains relatively consistent, with age between 40 and 80 contribute the greatest part of the total number of incidence, prevalence, and DALYs (Additional file: sFigure 2). Due to age restrictions, estimates for patients younger than 5 year were not available for rheumatoid arthritis (Additional file: sFigure 2-A, B, C), low back pain (Additional file: sFigure 2-G, H, I), and neck pain (Additional file: sFigure 2-J, K, L). In addition, the age categories of 1 to 30 and 1 to 15 had no cases of osteoarthritis (Additional file: sFigure 2-D, E, F) and gout (Additional file: sFigure 2-M, N, O), respectively. The number and age-standardized rate of incidence, prevalence, and DALYs was higher in females for rheumatoid arthritis, osteoarthritis, low back pain, and neck pain, but higher in males for gout (Additional file: sFigure 3).

### Association between disease burden and SDI

The absolute number in incidence, prevalence, and DALYs were positively correlated with the SDI level for five musculoskeletal disorders. The age-standardized rate of incidence, prevalence, and DALYs also positively correlated with the SDI level for rheumatoid arthritis, osteoarthritis, neck pain, and gout, but negatively correlated with the SDI level for low back pain (correlation coefficient − 0.807, − 0.808, − 0.810) (Additional file: sTable 3).

**Fig. 2:**
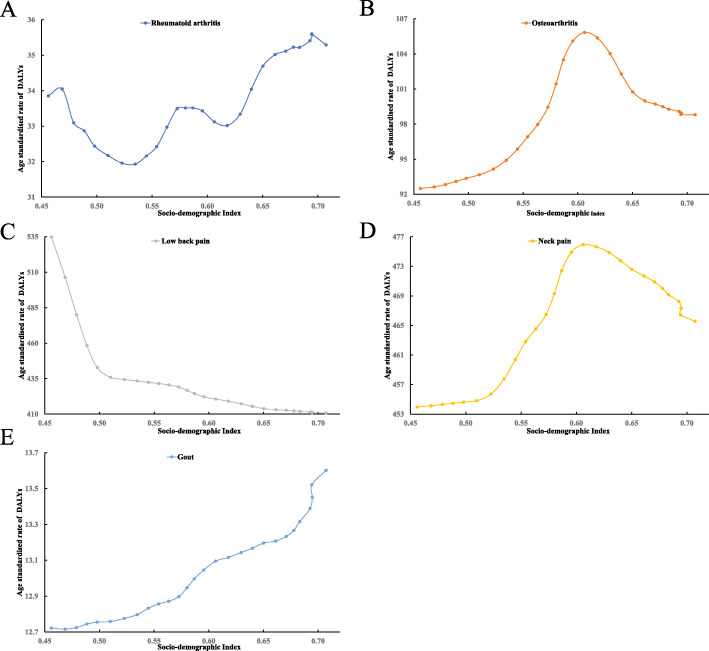
Correlation of age-standardized rate of DALYs with SDI for five major musculoskeletal disorders between 1990 and 2017. Correlation of age-standardized rate of DALYs with SDI for rheumatoid arthritis (**a**), osteoarthritis(**b**), low back pain(**c**), neck pain (**d**), and gout (**e**). SDI sociodemographic index

The non-linear associations between SDI and age-standardized rate of DALYs for rheumatoid arthritis, osteoarthritis, low back pain, neck pain, and gout are shown in Fig. 2. With the development of SDI, there was a zigzagged increasing trend of DALYs for rheumatoid arthritis (Fig. 2a) and curvilinear increasing for gout (Fig. 2e), but a curvilinear decreasing trend for low back pain (Fig. 2d). These results demonstrated that the expected value for DALYs for osteoarthritis (Fig. 2b) and neck pain (Fig. 2d) reaches the highest point when the SDI value is about 0.61. A similar trend was also observed for the age-standardized rate of incidence and prevalence for rheumatoid arthritis, osteoarthritis, low back pain, neck pain, and gout (Additional file: sFigure 4). The trend in age-standardized rate of DALYs was consistent with that of incidence and prevalence for osteoarthritis, low back pain, neck pain, and gout, but not for rheumatoid arthritis (Additional file: sFigure 4-A, B vs Fig. 2a).

### Temporal trend analysis of disease burden

Overall, there is a significant increasing trend for rheumatoid arthritis (Additional file: sFigure 5-A, B, C), osteoarthritis (Additional file: sFigure 6-A, B, C), neck pain (Additional file: sFigure 8- A, B, C), and gout (Additional file: sFigure 9-A, B, C), but a decreasing trend for low back pain (Additional file: sFigure 7-A, B, C) throughout the study period. According to the Joinpoint regression model, the trends in the annual age-standardized rate of incidence, prevalence, and DALYs are showed in Table 1. For the full 1990–2017 study period, the ranking of AAPC for age-standardized rate of incidence were rheumatoid arthritis (0.93, 95% CI 0.86, 1.00), gout (0.23, 95% CI 0.21, 0.24), osteoarthritis (0.14, 95% CI 0.09, 0.18), and neck pain (0.07, 95% CI 0.06, 0.08); for age-standardized rate of prevalence were rheumatoid arthritis (0.88, 95% CI 0.83, 0.93), gout (0.25, 95% CI 0.23, 0.27), osteoarthritis (0.24, 95% CI 0.19, 0.30), and neck pain (0.08, 95% CI 0.08, 0.09); but for age-standardized rate of DALYs were osteoarthritis (0.26, 95% CI 0.20, 0.31), gout (0.25, 95% CI 0.23, 0.27), rheumatoid arthritis (0.20, 95% CI 0.07, 0.34), and neck pain (0.09, 95% CI 0.07, 0.12), respectively (Table 1). However, the annual age-standardized rate of incidence (AAPC = − 0.91, 95% CI − 0.94, − 0.89), prevalence (AAPC = − 0.96, 95% CI − 0.98, − 0.93), and DALYs (AAPC = − 0.96, 95% CI − 0.98, − 0.93) for low back pain significantly decreased during the 1990–2017 period (Table 1).

**Table 1:** The AAPC and APC in age-standardized rate of incidence, prevalence, and DALYs for five major musculoskeletal disorders

	Year range	Incidence (95% CI)	Year range	Prevalence (95% CI)	Year range	DALYs (95% CI)
Average annual percent change (AAPC)						
Rheumatoid arthritis	1990–2017	0.93 (0.86 to 1.00)*	1990–2017	0.88 (0.83 to 0.93)*	1990–2017	0.20 (0.07 to 0.34)*
Osteoarthritis		0.14 (0.09 to 0.18)*		0.24 (0.19 to 0.30)*		0.26 (0.20 to 0.31)*
Low back pain		− 0.91 (− 0.94 to − 0.89)*		− 0.96 (− 0.98 to − 0.93)*		− 0.96 (− 0.98 to − 0.93)*
Neck pain		0.07 (0.06 to 0.08)*		0.08 (0.08 to 0.09)*		0.09 (0.07 to 0.12)*
Gout		0.23 (0.21 to 0.24)*		0.25 (0.23 to 0.27)*		0.25 (0.23 to 0.27)*
Annual percent change (APC)						
Rheumatoid arthritis	1990–2000	0.92 (0.86 to 0.98)*	1990–2000	0.79 (0.75 to 0.83)*	1990–1995	− 1.19 (− 1.85 to − 0.52)*
	2000–2005	− 0.81 (− 1.05 to − 0.56)*	2000–2005	− 1.00 (− 1.17 to − 0.83)*	1995–2007	0.39 (0.19 to 0.60)*
	2005–2010	3.14 (2.88 to 3.40)*	2005–2010	3.26 (3.07 to 3.45)*	2007–2010	1.43 (− 1.68 to 4.63)
	2010–2017	0.64 (0.53 to 0.75)*	2010–2017	0.69 (0.61 to 0.77)*	2010–2017	0.18 (− 0.26 to 0.63)
Osteoarthritis	1990–1998	0.35 (0.28 to 0.42)*	1990–1998	0.37 (0.30 to 0.44)*	1990–1998	0.41 (0.34 to 0.48)*
	1998–2005	1.52 (1.42 to 1.63)*	1998–2005	1.60 (1.49 to 1.72)*	1998–2005	1.61 (1.50 to 1.72)*
	2005–2010	−1.80 (−1.98 to − 1.61)*	2005–2010	−1.27 (− 1.47 to − 1.06)*	2005–2010	− 1.28 (− 1.48 to − 1.08)*
	2010–2017	− 0.09 (− 0.17 to − 0.02)*	2010–2017	− 0.17 (− 0.25 to − 0.09)*	2010–2017	− 0.16 (− 0.25 to − 0.07)*
Low back pain	1990–1994	− 4.50 (− 4.61 to − 4.40)*	1990–1994	− 4.71 (− 4.81 to − 4.60)*	1990–1994	− 4.74 (− 4.84 to − 4.64)*
	1994–2001	− 0.33 (− 0.38 to − 0.28)*	1994–2001	− 0.36 (− 0.41 to − 0.31)*	1994–2001	− 0.32 (− 0.38 to − 0.26)*
	2001–2008	− 0.42 (− 0.47 to − 0.36)*	2001–2009	− 0.42 (− 0.46 to − 0.38)*	2001–2009	− 0.44 (− 0.49 to − 0.40)*
	2008–2017	− 0.12 (− 0.15 to − 0.09)*	2009–2017	− 0.09 (− 0.13 to − 0.06)*	2009–2017	− 0.09 (− 0.13 to − 0.05)*
Neck pain	1990–1996	0.02 (0.00 to 0.04)*	1990–1996	0.03 (0.01 to 0.04)*	1990–1993	0.03 (− 0.07 to 0.12)
	1996–2004	0.35 (0.33 to 0.36)*	1996–2001	0.48 (0.45 to 0.51)*	1993–1996	0.09 (−0.10 to 0.28)
	2004–2007	0.06 (−0.04 to 0.16)	2001–2005	0.52 (0.48 to 0.57)*	1996–2005	0.50 (0.48 to 0.52)*
	2007–2017	− 0.12 (− 0.12 to − 0.11)*	2005–2017	− 0.20 (− 0.20 to − 0.19)*	2005–2017	− 0.20 (− 0.21 to − 0.19)*
Gout	1990–2000	0.05 (0.03 to 0.06)*	1990–2000	0.08 (0.06 to 0.09)*	1990–2000	0.12 (0.10 to 0.14)*
	2000–2005	0.41 (0.34 to 0.47)*	2000–2005	0.43 (0.36 to 0.50)*	2000–2005	0.37 (0.29 to 0.45)*
	2005–2012	0.22 (0.18 to 0.25)*	2005–2012	0.21 (0.18 to 0.25)*	2005–2012	0.18 (0.14 to 0.22)*
	2012–2017	0.41 (0.36 to 0.46)*	2012–2017	0.47 (0.42 to 0.52)*	2012–2017	0.50 (0.45 to 0.55)*

*AAPC* average annual percent changes, *APC* annual percent change, *CI* confidence interval. *The AAPC or APC is significantly different from zero at the alpha = 0.05 level

For APC in rheumatoid arthritis, the incidence and prevalence present as a small increasing, small decreasing, large increasing, and small increasing trend during the 1990–2000, 2000–2005, 2005–2010, 2010–2017 period, respectively, while the DALYs presents as a large decreasing, small increasing, large increasing, small increasing trend during the 1990–1995, 1995–2007, 2007–2010, 2010–2017 period, respectively (Table 1, Additional file: sFigure 5-A, B, C). For APC in osteoarthritis, the incidence, prevalence, and DALYs present as a small increasing, large increasing, large decreasing, small decreasing trend during the 1990–1998, 1998–2005, 2005–2010, 2010–2017 period, respectively (Table 1, Additional file: sFigure 6-A, B, C). For APC in low back pain, the incidence presents as large decreasing and three small decreasing trends during the 1990–1994, 1994–2001, 2001–2008, 2008–2017 period, respectively; prevalence and DALYs present as large decreasing and three small decreasing trends during the 1990–1994, 1994–2001, 2001–2009, 2009–2017 period, respectively (Table 1, Additional file: sFigure 7-A, B, C). For APC in neck pain, the incidence presents a small increasing, small increasing, stable, and small decreasing trend during the 1990–1996, 1996–2004, 2004–2007, 2007–2017 period; the prevalence presents a three-small increasing and one small decreasing trend during the 1990–1996, 1996–2001, 2001–2005, 2005–2017 period; the DALYs presents a two stable, small increasing, small decreasing trend during the 1990–1993, 1993–1996, 1996–2005, 2005–2017 period, respectively (Table 1, Additional file: sFigure 8-A, B, C). For APC in gout, the incidence, prevalence, and DALYs present as four small increasing trends during the 1990–2000, 2000–2005, 2005–2012, 2012–2017 period, respectively (Table 1, Additional file: sFigure 9-A, B, C).

### Temporal trend analysis of risk factor

**Fig. 3:**
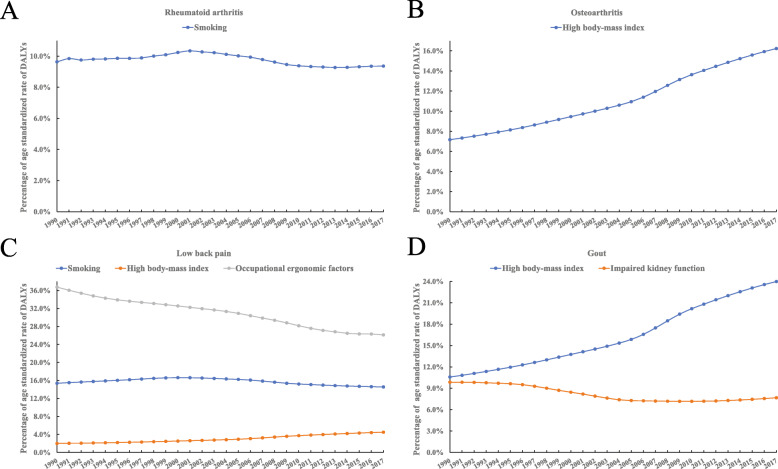
Temporal trends of the population attributable fraction of age-standardized rate DALYs for rheumatoid arthritis, osteoarthritis, low back pain, and gout between 1990 and 2017. Smoking for rheumatoid arthritis (**a**); high body-mass index for osteoarthritis (**b**); smoking, high body-mass index, occupational ergonomic factors for low back pain (**c**); high body-mass index, impaired kidney function for gout (**d**). All data are expressed as the percentage of age-standardized rate of DALYs per 100,000 persons

The analysis of annual PAF due to risk factors indicated a significant consistent decrement of smoking (AAPC − 0.22, 95% CI − 0.24, − 0.19) and occupational ergonomic factors (AAPC − 1.25, 95% CI − 1.29, − 1.21) for low back pain (Fig. 3c), and impaired kidney function (AAPC − 0.95, 95% CI − 1.00, − 0.90) for gout (Fig. 3d), while a consistent significant increment of high BMI for osteoarthritis (AAPC 3.10, 95% CI 3.03, 3.17, Fig. 3b), low back pain (AAPC 3.07, 95% CI 2.99, 3.14, Fig. 3c), and gout (AAPC 3.12, 95% CI 3.04, 3.20, Fig. 3d). Although there is an overall decrement of smoking for rheumatoid arthritis (AAPC − 0.14, 95% CI − 0.24, − 0.04), it increased from 1990, reached the peak at 2001, and then turned to decrease (Fig. 3a) (Table 2).

**Table 2:** The AAPC and APC of population attributable fractions (PAFs) for DALYs related to rheumatoid arthritis, osteoarthritis, low back pain, and gout due to risk factors

Disease: risk factor	AAPC	APC			
Rheumatoid arthritis	1990–2017	1990–1997	1997–2002	2002–2011	2011–2017
Smoking	− 0.14 (− 0.24, − 0.04)*	0.29 (0.13 to 0.46)*	0.91 (0.51 to 1.32)*	− 1.23 (− 1.37 to − 1.09)*	0.12 (− 0.10 to 0.33)
Osteoarthritis	1990–2017	1990–1994	1994–2005	2005–2010	2010–2017
High body-mass index	3.10 (3.03, 3.17)*	2.53 (2.21 to 2.86)*	2.98 (2.90 to 3.05)*	4.69 (4.43 to 4.94)*	2.50 (2.41 to 2.59)*
Low back pain	1990–2017	1990–2000	2000–2005	2005–2010	2010–2017
Smoking	− 0.22 (− 0.24, − 0.19)*	0.82 (0.81 to 0.83)*	− 0.51 (− 0.55 to − 0.47)*	− 1.38 (− 1.43 to − 1.34)*	− 0.64 (− 0.66 to − 0.62)*
Low back pain	1990–2017	1990–1994	1994–2005	2005–2010	2010–2017
High body-mass index	3.07 (2.99, 3.14)*	1.62 (1.41 to 1.83)*	2.89 (2.85 to 2.94)*	5.21 (5.05 to 5.38)*	2.65 (2.59 to 2.71)*
Low back pain	1990–2017	1990–1994	1994–2004	2004–2013	2013–2017
Occupational ergonomic factors	− 1.25 (− 1.29, − 1.21)*	− 1.74 (− 1.89 to − 1.58)*	− 0.83 (− 0.88 to − 0.79)*	− 1.80 (− 1.86 to − 1.75)*	− 0.56 (− 0.74 to − 0.39)*
Gout	1990–2017	1990–2005	2005–2009	2009–2013	2013–2017
High body-mass index	3.12 (3.04, 3.20)*	2.74 (2.70 to 2.78)*	5.48 (5.08 to 5.87)*	3.20 (2.86 to 3.54)*	2.12 (1.92 to 2.32)*
Gout	1990–2017	1990–1996	1996–2005	2005–2012	2012–2017
Impaired kidney function	− 0.95 (− 1.00, − 0.90)*	− 0.56 (− 0.62 to − 0.50)*	− 3.11 (− 3.14 to − 3.07)*	− 0.05 (− 0.10 to 0.01)	1.29 (1.21 to 1.36)*

*AAPC* average annual percent changes, *APC* annual percent change, *CI* confidence interval. *The AAPC or APC is significantly different from zero at the alpha = 0.05 level

For APC in rheumatoid arthritis, smoking presents as a small increasing, small increasing, large decreasing, stable trend during the 1990–1997, 1997–2002, 2002–2011, 2011–2017 period (Table 2, Fig. 3a). For APC in osteoarthritis, high body-mass index presents as four large increasing trends during the 1990–1994, 1994–2005, 2005–2010, 2010–2017 period (Table 2, Fig. 3b). For APC in low back pain, smoking presents as a small increasing, small decreasing, large decreasing, small decreasing trend during the1990–2000, 2000–2005, 2005–2010, 2010–2017 period; high body-mass index presents as four large increasing trends during the 1990–1994, 1994–2005, 2005–2010, 2010–2017; occupational ergonomic factors present as large decreasing, small decreasing, large decreasing, small decreasing trend during the 1990–1994, 1994–2004, 2004–2013, 2013–2017 period (Table 2, Fig. 3c). For APC in gout, high body-mass index presents as four large increasing trends during the 1990–2005, 2005–2009, 2009–2013, 2013–2017 period; impaired kidney function presents as small decreasing, large decreasing, small decreasing, large increasing trend during the1990–1996, 1996–2005, 2005–2012, 2012–2017 period (Table 2, Fig. 3d).

### Overall burden ranking in group of 20 countries

**Fig. 4:**
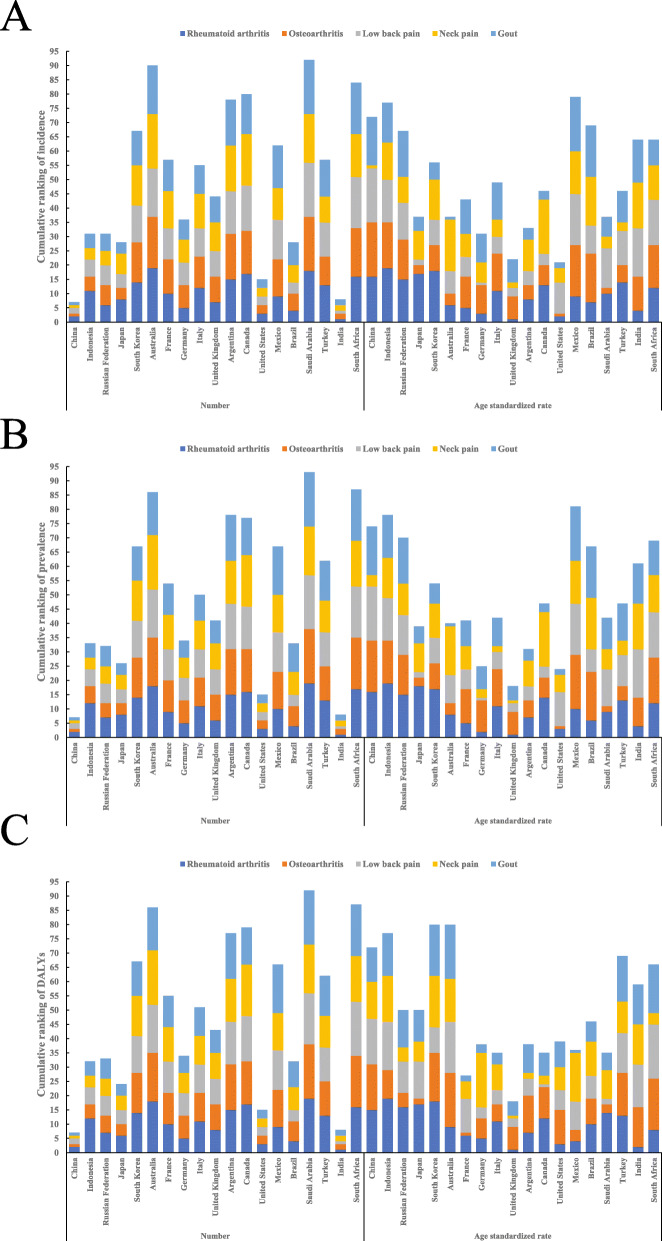
Cumulative ranking of incidence, prevalence, DALYs for 5 major musculoskeletal disorder in 19 of Group 20 countries except European Union. Cumulative ranking of number (left) and age standardized rate (right) of incidence for 5 major musculoskeletal disorder in 19 of Group 20 countries except European Union (**a**) ; Cumulative ranking of number (left) and age standardized rate (right) of prevalence for 5 major musculoskeletal disorder in 19 of Group 20 countries except European Union (**b**) ; Cumulative ranking of number (left) and age standardized rate (right) of DALYs for 5 major musculoskeletal disorder in 19 of Group 20 countries except European Union (**c**). The cumulative ranking (5-95) was sum of rank of incidence, prevalence, DALYs for each country according to either the number or age standardized rate

Comparing the burden of rheumatoid arthritis, osteoarthritis, low back pain, neck pain, and gout in China with the 19 member countries of Group 20, except European Union, China was ranked 2nd, 1st, 2nd, 1st, and 1st in terms of the number of incidence; ranked 2nd, 1st, 2nd, 1st, and 1st in terms of the number of prevalence; and ranked 2nd, 1st, 2nd, 1st, and 1st in terms of the number of DALYs; China was ranked 16th, 19th, 19th, 1st, and 17th in terms of age-standardized rate of incidence; 16th, 18th, 19th, 4th, and 17th in terms of age-standardized rate of prevalence; and 15th, 16th, 16th, 13th, and 12th in terms of age-standardized rate of DALYs, respectively (Fig. 4, Additional file: sTable 4).

## Discussion

### Main finding

The disease burden due to five major musculoskeletal disorders increased with age, while age between 40 and 80 contributes the greatest part. The incidence, prevalence, and DALYs between ages 40 and 80 were 70.1%, 77.1%, and 74.3% of total incidence, prevalence, and DALYs due to five major musculoskeletal disorders, respectively. Therefore, population aging will probably be the main drivers of the musculoskeletal burden, which has become increasingly important in terms of potential policy implications, such as healthcare provisions and extending retirement ages, because increasing life expectancy in China over these years could offset the relatively small population increases due to the loosen of population control measures in China. On the one hand, the population increase will be relatively small although China’s one-child policy was replaced by a universal two-child policy in 2015 [13]. On the other hand, life expectancy at age 60 years was 19.39 for males and 22.81 for females [3].

According to the Joinpoint regression analysis, the decrease in age-standardized rate of incidence, prevalence, and DALYs related to low back pain suggests effective control, but an increase related to rheumatoid arthritis, osteoarthritis, neck pain, and gout represents an ongoing challenge. This suggests the importance of increased investment in the prevention and treatment of musculoskeletal disease in rheumatoid arthritis, osteoarthritis, neck pain, and gout. The increase for rheumatoid arthritis, osteoarthritis, neck pain, and gout potentially explained by increased investment in health professionals and hospitals. The number of hospitals increased from 14,377 in 1990 to 31,056 in 2017; the number of beds increased from 29,254,000 in 1990 to 79,403,000 in 2017; and the number of health professionals per thousand population increased from 3.45 in 1990 to 6.47 in 2017 [14].

The discovery of the “pre-rheumatoid arthritis” stage of seropositive disease and an ability to predict development of future rheumatoid arthritis help to identify ways to delay or prevent the onset of clinically apparent rheumatoid arthritis [15]. Thus, it is reasonable for subjects at-risk for rheumatoid arthritis to periodically seek medical attention if joint symptoms develop or worsen. Osteoarthritis is known as a slowly progressive disease with irreversible structural change, but there are no validated early-stage diagnostic criteria of osteoarthritis to adopt proactive management and sensitive outcome measures to test intervention in early-stage disease [16]. Most acute episodes of neck pain resolve spontaneously, but more than a third of affected people still have low-grade symptoms or recurrences for long time, with genetics and psychosocial factors being risk factors for persistence [17]. Thus, the identification of phenotype of neck pain with either an identified pathoanatomical basis or defined through clinical reasoning will probably determine which treatment target should we approached [17]. Besides, we have a limited understanding of how to manage the neck pain, especially for children and older people, because they are usually excluded from studies of intervention [18]. Although the associations between novel comorbid diseases and gout have been identified, and understanding the relationships between different comorbidities in individuals with gout has improved, worrying increase, uptake of, adherence with, and persistence with definitive, curative urate-lowering therapies continue to be poor [19]. In addition, a growing portion of the working-age population spends most of its time in office environments, leading to increased burden of low back pain and neck pain, which suggests the need for relevant policies to prevent this occupational hazard [20].

The positive link between the number of incidence, prevalence, and DALYs from 5 musculoskeletal disorders and SDI due to societal development might be due to increased access to medical resources, such as healthcare facility locations and health insurance. With the development of socioeconomic status, as measured by the SDI, there is an increasing trend of age-standardized rate of disease burden from rheumatoid arthritis, osteoarthritis, neck pain, and gout, but a decreasing trend from low back pain. These results show that the higher social classes were more susceptible to rheumatoid arthritis, osteoarthritis, neck pain, and gout than lower social classes. The apparent paradox between number and age-standardized rate of disease burden from low back pain is reasonable and explicable in terms of socioeconomic development due to increasing awareness of physical activity and healthy dietary patterns.

The contribution of high BMI (osteoarthritis, low back pain, gout) is continuing to increase while the effect of smoking (rheumatoid arthritis, low back pain), occupational ergonomic factors (low back pain), and impaired kidney function (gout) continued to decrease. The potential effect of smoking, occupational ergonomic factors, and impaired kidney function seems decreasing, but more attention should be paid to the effect of high BMI. A Mendelian randomization study in the UK Biobank indicated that BMI exerts a major causal effect on the risk of osteoarthritis at weight-bearing joints [21]. Another Mendelian randomization study found a significant causal effect of BMI on both back pain and chronic back pain [22]. Similarly, overall obesity was identified as a causal factor for gout and is associated with higher serum urate concentrations [23]. Between 1990 and 2017, exposure to high BMI is rising at the national level [1]. High BMI was also causally associated with an increased risk of rheumatoid arthritis [24]. Unfortunately, GBD 2017 study only quantify the contribution of smoking for rheumatoid arthritis, but do not quantify the contribution of high BMI for rheumatoid arthritis [9]. Therefore, population-wide initiatives targeting high BMI may mitigate the burden of five major musculoskeletal disorders [25].

Although China ranked top 2 in terms of total numbers of DALYs related to rheumatoid arthritis, osteoarthritis, low back pain, neck pain, and gout, and ranked last 7 in terms of age-standardized rate of DALYs among 19 member countries of the G20, the burden due to five major musculoskeletal disease continues to increase due to population aging and population growth despite a substantial health investment. The burden of five major musculoskeletal disorders in China was somewhat less than that in India and was worse than those in other member countries of the G20. This was to be expected, taking China’s large population into account. However, it is worth noting that China has a relatively lower age-standardized rate of incidence, prevalence, and DALYs for five major musculoskeletal disorders compared with other member countries of the G20, such as the USA and UK. Although life expectancy at birth in China is quite close to the USA (76.09 for male and 81.09 for female) and UK (79.18 for male and 82.72 for female), China has a significant higher increase in life expectancy over the past 28 years compared with other countries, which may have led to a lower prevalence of age-related musculoskeletal disorders [3]. This is quite concerning, as there might be unfilled medical gap in controlling musculoskeletal diseases.

### Study strength

The study has strengths. To our knowledge, this study provides the most comprehensive estimates of the burden due to the five major musculoskeletal disorders in China, with attention paid to temporal trends and demonstrate the importance of disease and risk factor-specific investment in the prevention and control of musculoskeletal diseases in China. Besides, the GBD study uses unified and standard methodology in data analytical techniques, making these estimates comparable across time. Moreover, the temporal trends are perhaps the most important observation regarding the disease burden due to the five musculoskeletal diseases in China. Furthermore, not only alteration during the entire period (assessed by AAPC), but also each segmental period (assessed by APC) was determined using the Joinpoint regression model. Lastly, using SDI in our analysis has helped to provide important evidence for tailored, disease-specific policy development and interventions, helping governments to facilitate priority setting. For example, the government could increase public health investment for people with musculoskeletal disorders at an early stage to attenuate disease severity at the population level, particularly for preventable burden in younger adults.

### Study limitation

The primary limitation of data sources in the present study is the unavailability of high-quality data in each time point and the results presented here were mainly derived from modeled data through the processes in DisMod-MR 2.1 [3, 4, 6, 9]. True population-based national data on annual incidence and point prevalence of five musculoskeletal disorders were available from very few countries; thus, the pattern and trend of five major musculoskeletal disorders in China from 1990 to 2017 were produced from modeled data in DisMod-MR 2.1 driven by statistical modeling of covariates [4, 6]. As such, the temporal trend estimates in China should be interpreted with caution. Greater inclusion of musculoskeletal conditions in national health data collections and importing them in the model is therefore encouraged to ensure the capture of more representative and accurate data. National musculoskeletal disorder collection strategy could not only be used to monitor the burden of musculoskeletal disorders, but also to evaluate the long-term efficiency and effectiveness of drugs and treatment strategy, such as biologic agents and treat to target strategy. Besides, although DisMod-MR 2.1 is designed to capture uncertainty and synthesized sparse and heterogeneous epidemiological data, but it may not fully represent the uncertainty interval around estimates, particularly in locations with sparse or absent data, and cannot simultaneously solve temporal trend over age and time [6].

The main source of bias in the data sources is that GBD 2017 assumed that the available in-hospital and out-of-hospital data are representative of the whole population in China in each time point, which might underestimate the disease burden of five musculoskeletal disorders in the vast remote and poor rural areas, because there is an unbalanced distribution of medical resources between urban and rural areas although the accessibility to medical sources in rural areas was greatly improved during the past three decades [1]. In addition, differences in the quantity and quality of data inputted into the statistical model in different years could affect the accuracy of the estimated temporal trends due to the iteration of diagnostic technology, updated classification criteria, and number and level of rheumatologists over time.

Our study also has general limitations. First, as part of the GBD 2017 study, the study is subject to all the limitations of the GBD 2017 studies and the quantity and quality of data available are still limited, which could affect the accuracy of the estimated burden [2–4, 6, 9, 26, 27]. Second, the group of other musculoskeletal disorders are excluded from the study due to a wide range of specific diseases. Third, the estimated five major musculoskeletal disorder burden in GBD 2017 may be incomplete due to failure to perform province-level, and urban-rural stratification of results, which are beneficial for understanding disparities to direct appropriate health policy and programs [1]. The temporal trend in province level and between urban and rural areas would be more reliable and precise if systematic efforts were made to fill the data gaps. Further improvements in data definition, measurement, collection, and modeling strategy for age and time might lead to more stable estimates in temporal trend and decrease potential out-of-sample modeling.

## Conclusion

There are remarkably complex temporal patterns in disease burden and risk factors for five major musculoskeletal disorders across past three decades. Population-wide initiatives targeting high body-mass index may mitigate the burden of musculoskeletal disorders.

## Supplementary Information

**Additional file 1: sFigure 1.** Rate of incidence and prevalence for 5 major musculoskeletal disorders at all ages.

**Additional file 2: sFigure 2.** Number of incidence, prevalence, DALYs for 5 major musculoskeletal disorders at all ages.

**Additional file 3: sFigure 3.** Number and age standardized rate of incidence, prevalence, DALYs for 5 major musculoskeletal disorders by gender.

**Additional file 4: sFigure 4.** Correlation of age-standardized rate of incidence and prevalence with SDI for five major musculoskeletal disorder between 1990 and 2017.

**Additional file 5: sFigure 5.** Age standardized rate of incidence, prevalence, DALYs for rheumatoid arthritis between 1990 and 2017.

**Additional file 6: sFigure 6.** Age standardized rate of incidence, prevalence, DALYs for osteoarthritis between 1990 and 2017.

**Additional file 7: sFigure 7.** Age standardized rate of incidence, prevalence, DALYs for low back pain between 1990 and 2017.

**Additional file 8: sFigure 8.** Age standardized rate of incidence, prevalence, DALYs for neck pain between 1990 and 2017.

**Additional file 9: sFigure 9.** Age standardized rate of incidence, prevalence, DALYs for gout between 1990 and 2017.

**Additional file 10: sTable 1.** Number and age-standardized rates of incidence, prevalence, DALYs for five major musculoskeletal diseases in China, 2017.

**Additional file 11: sTable 2.** Population attributable fractions (PAFs) for YLDs, DALYs, and deaths related to rheumatoid arthritis, osteoarthritis, low back pain, Gout due to risk factors in China (2017).

**Additional file 12: sTable 3.** Correlation between SDI and incidence, prevalence and DALYs.

**Additional file 13: sTable 4.** The value and ranking of five major musculoskeletal disorders for 19 of Group 20 countries except European Union.

## Data Availability

The datasets analyzed during the current study are available in the https://gbd2017.healthdata.org/gbd-search/.

## Abbreviations

AAPC: Average annual percent change
APC: Annual percent change
BMI: Body-mass index
CI: Confidence interval
DALYs: Disability-adjusted life years
G20: Group of 20
GBD: Global Burden of Disease
ICD: International Classification of Diseases
PAF: Population attributable fraction
SDG: Sustainable development goals
SDI: Sociodemographic index
UI: Uncertainty intervals
YLD: Years lived with disability
