# Supplementary figures and images for: Pattern and trend of five major musculoskeletal disorders in China from 1990 to 2017: findings from the Global Burden of Disease Study 2017
Source: BMC Med. 2021 Feb 4;19:34. doi: 10.1186/s12916-021-01905-w (PMC7860632; doi:10.1186/s12916-021-01905-w)

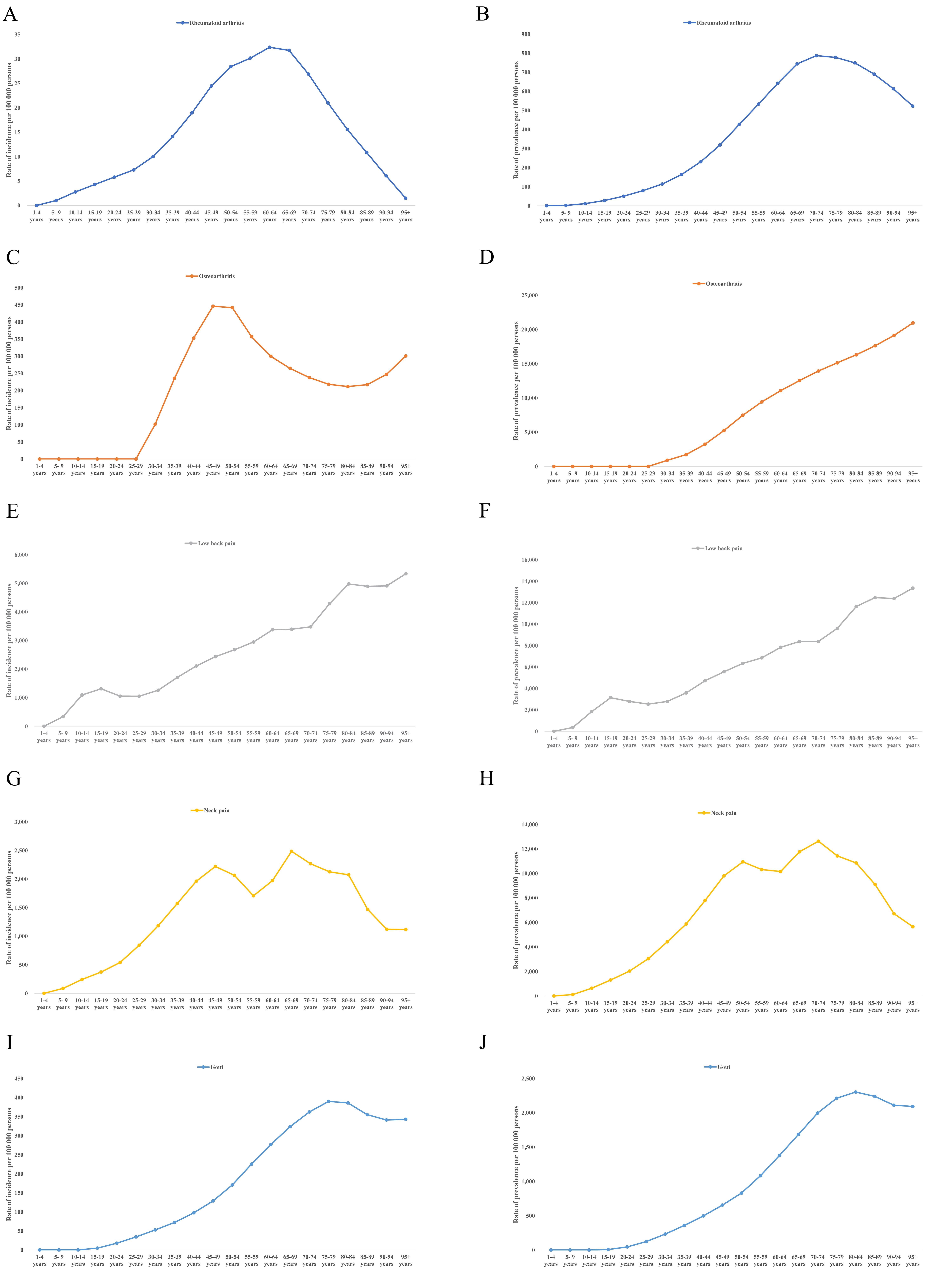

Supplement: Supplementary file 1 — Additional file 1: sFigure 1. Rate of incidence and prevalence for 5 major musculoskeletal disorders at all ages. [file 12916_2021_1905_MOESM1_ESM.tif]

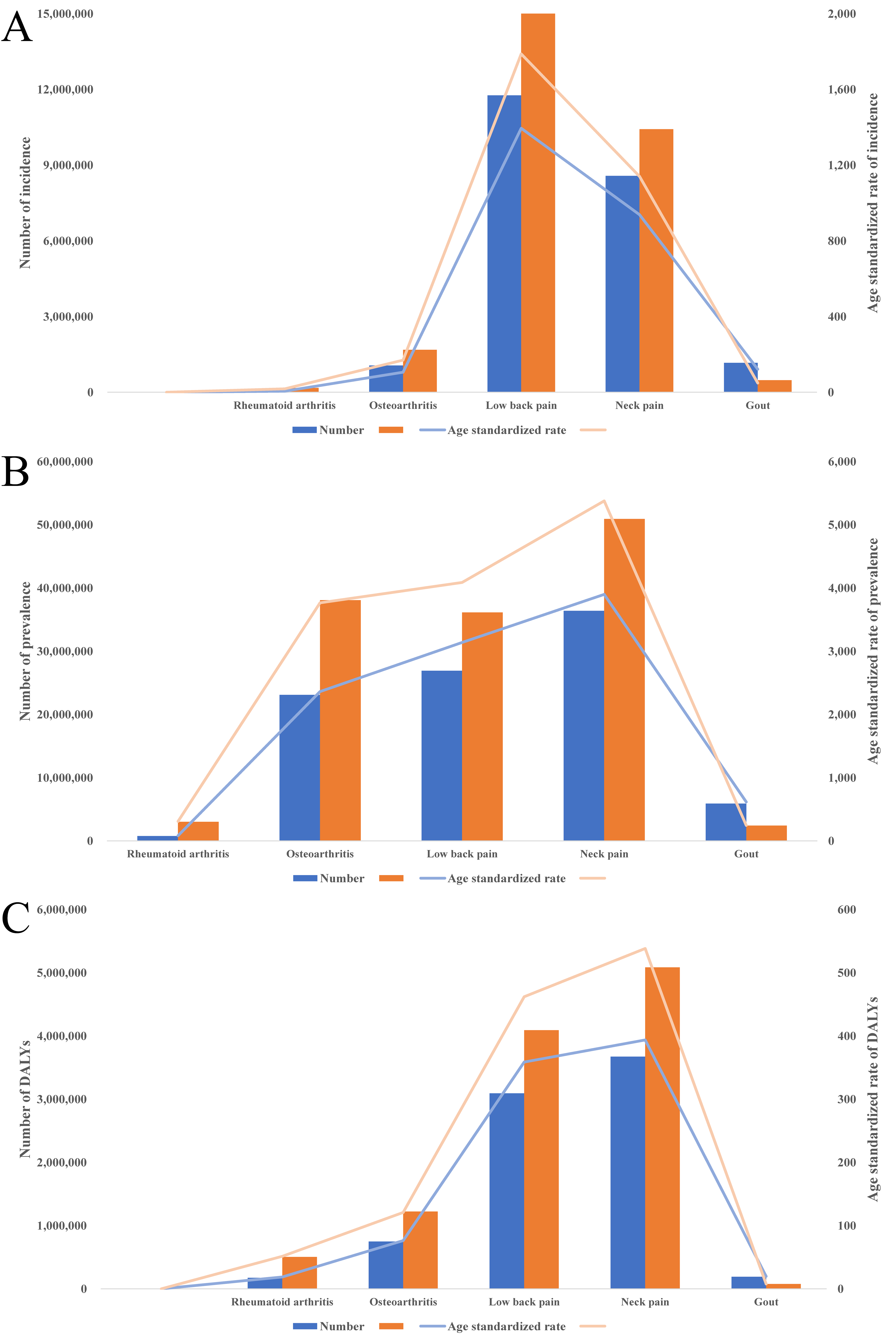

Supplement: Supplementary file 3 — Additional file 3: sFigure 3. Number and age standardized rate of incidence, prevalence, DALYs for 5 major musculoskeletal disorders by gender. [file 12916_2021_1905_MOESM3_ESM.tif]

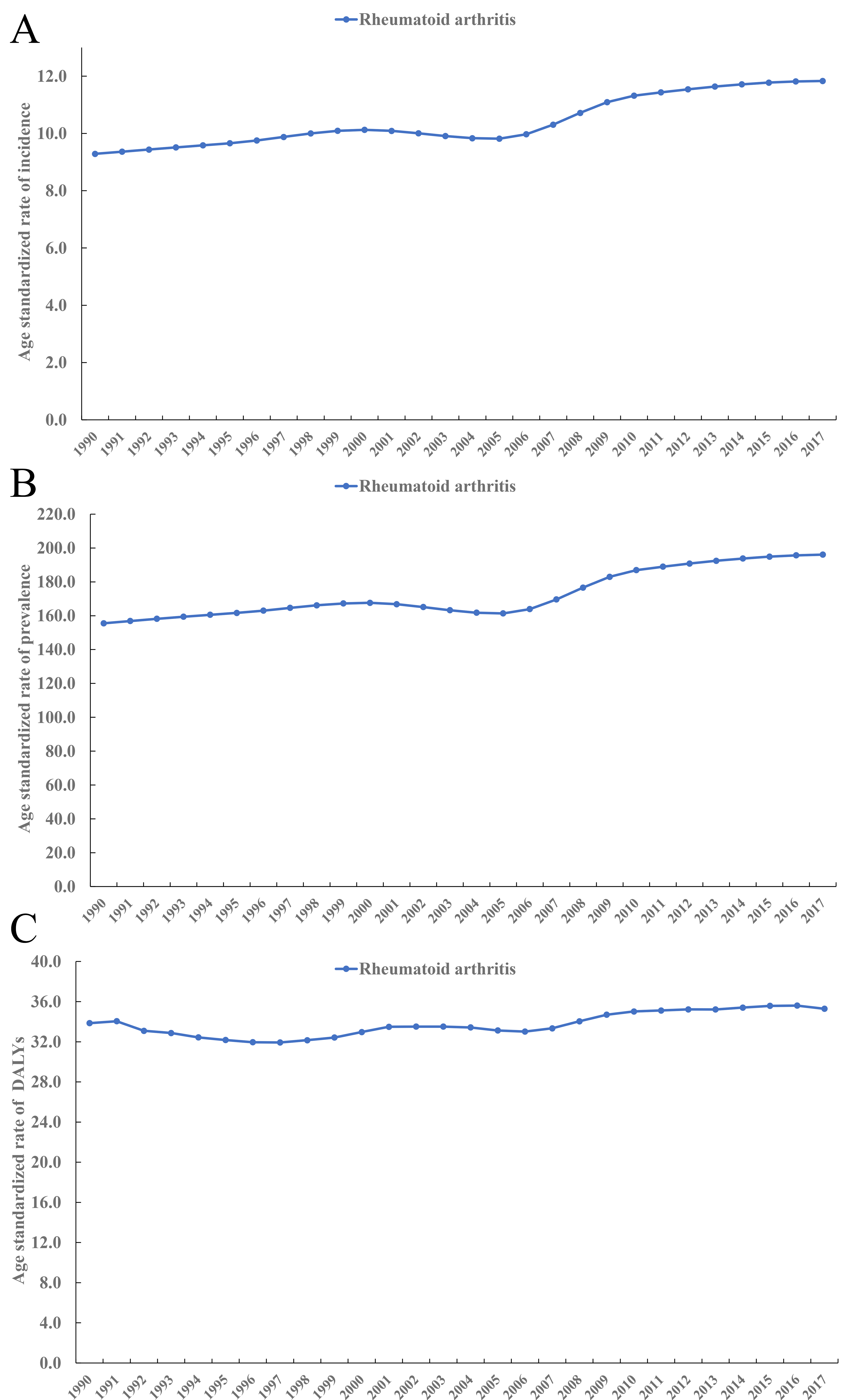

Supplement: Supplementary file 5 — Additional file 5: sFigure 5. Age standardized rate of incidence, prevalence, DALYs for rheumatoid arthritis between 1990 and 2017. [file 12916_2021_1905_MOESM5_ESM.tif]

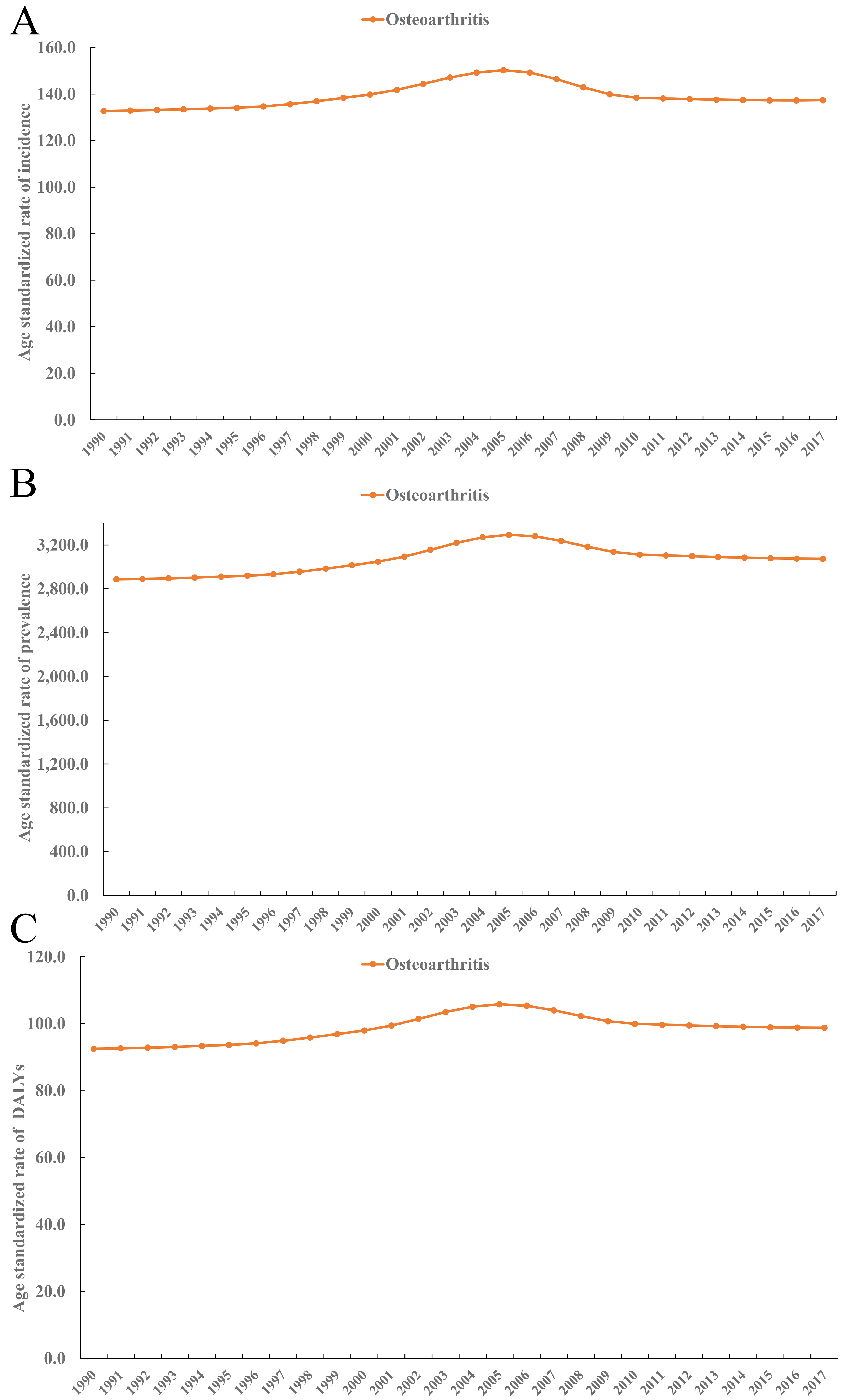

Supplement: Supplementary file 6 — Additional file 6: sFigure 6. Age standardized rate of incidence, prevalence, DALYs for osteoarthritis between 1990 and 2017. [file 12916_2021_1905_MOESM6_ESM.tif]

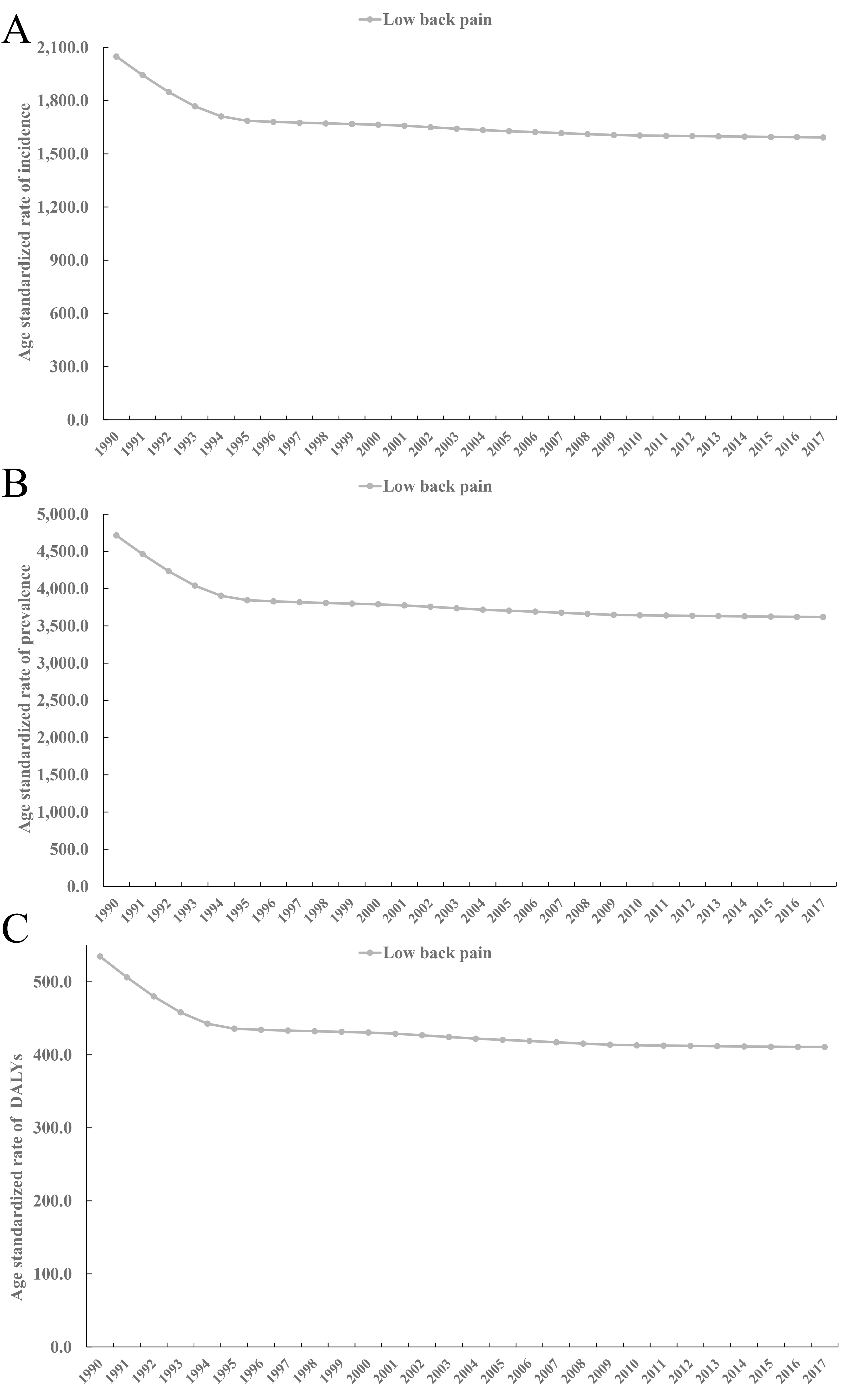

Supplement: Supplementary file 7 — Additional file 7: sFigure 7. Age standardized rate of incidence, prevalence, DALYs for low back pain between 1990 and 2017. [file 12916_2021_1905_MOESM7_ESM.tif]

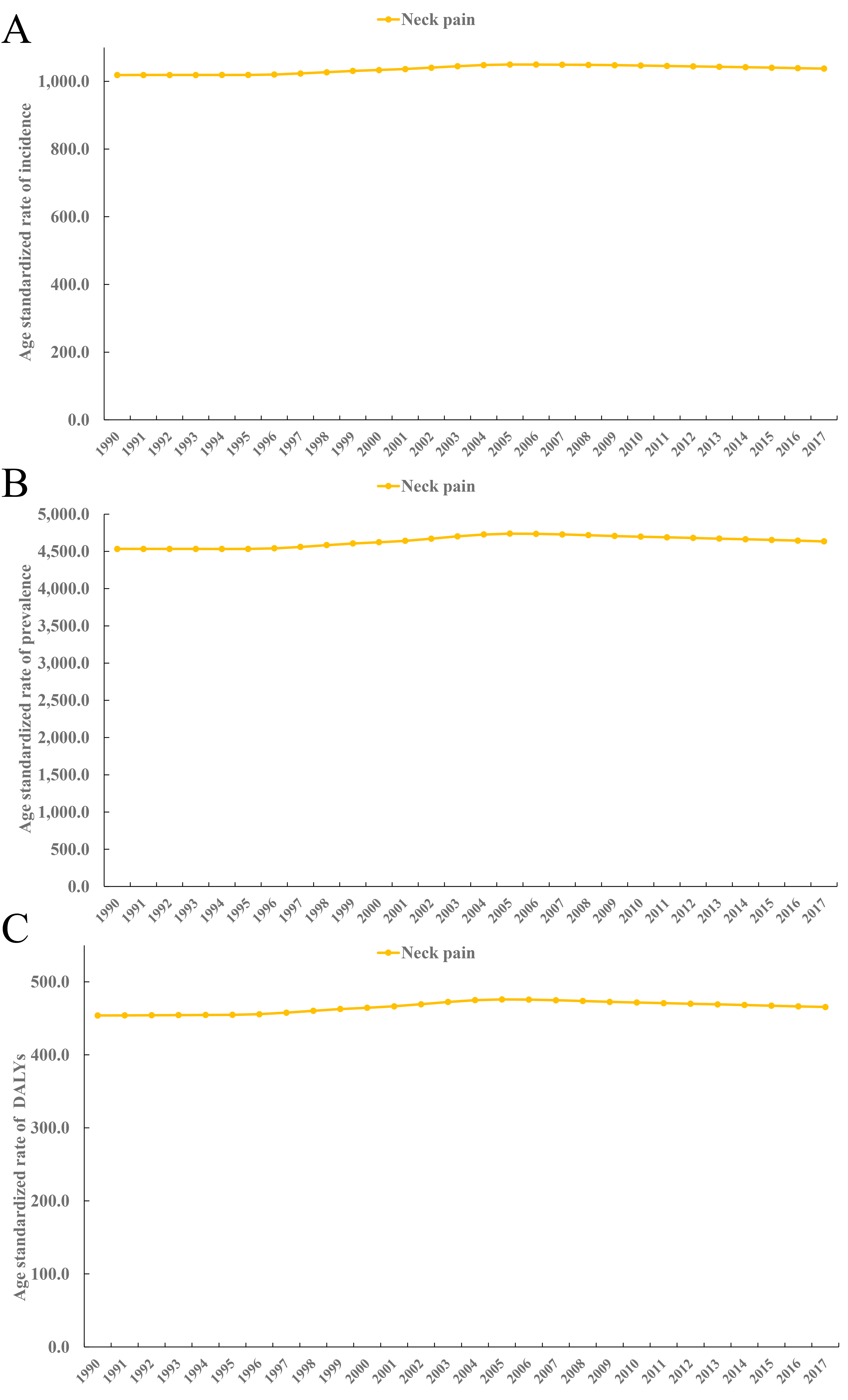

Supplement: Supplementary file 8 — Additional file 8: sFigure 8. Age standardized rate of incidence, prevalence, DALYs for neck pain between 1990 and 2017. [file 12916_2021_1905_MOESM8_ESM.tif]

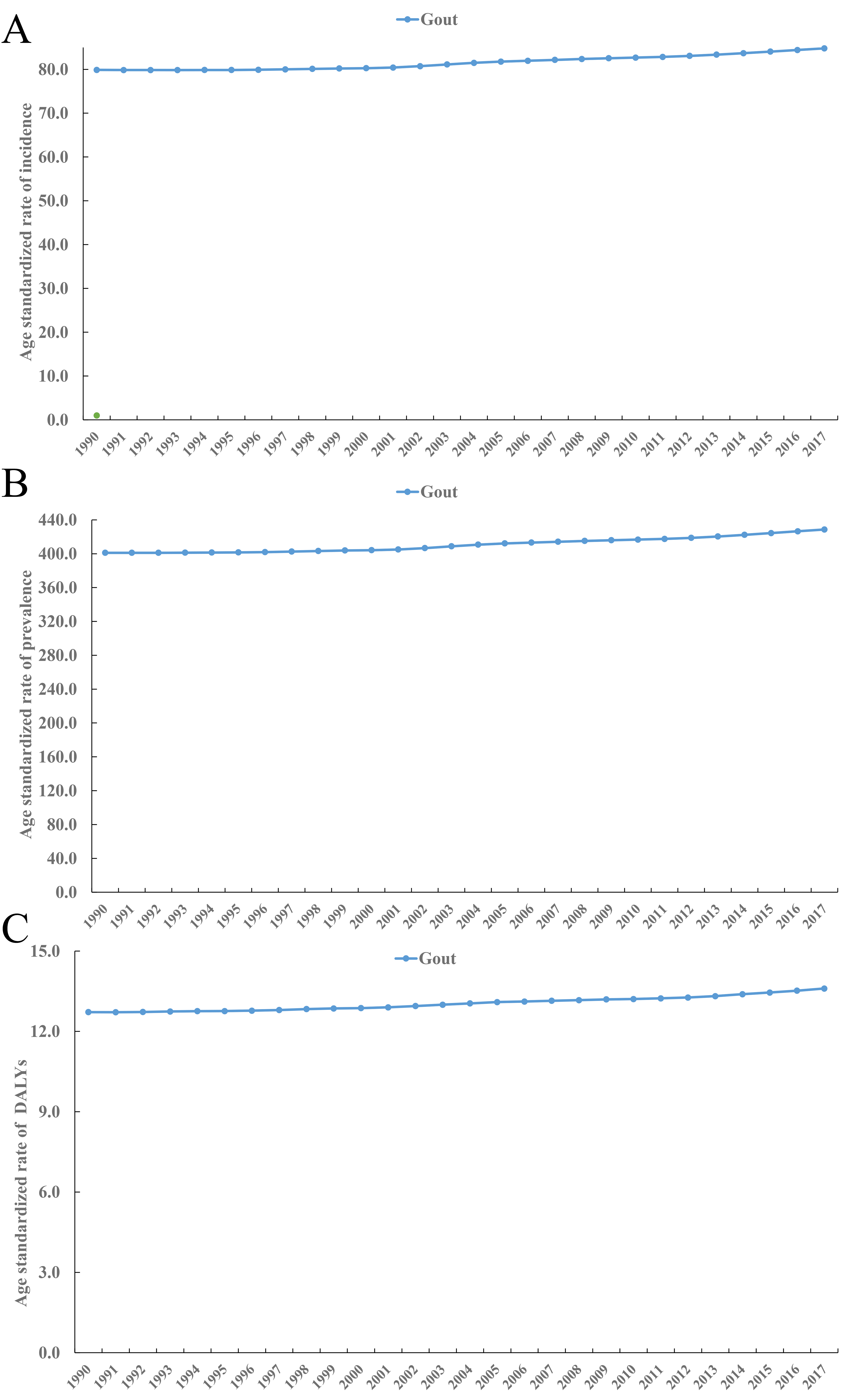

Supplement: Supplementary file 9 — Additional file 9: sFigure 9. Age standardized rate of incidence, prevalence, DALYs for gout between 1990 and 2017. [file 12916_2021_1905_MOESM9_ESM.tif]
