# Supplementary material for: Pattern and trend of five major musculoskeletal disorders in China from 1990 to 2017: findings from the Global Burden of Disease Study 2017
Source: BMC Med. 2021 Feb 4;19:34. doi: 10.1186/s12916-021-01905-w (PMC7860632; doi:10.1186/s12916-021-01905-w)
Supplement: Supplementary file 10 — Additional file 10: sTable 1. Number and age-standardized rates of incidence, prevalence, DALYs for five major musculoskeletal diseases in China, 2017. [file 12916_2021_1905_MOESM10_ESM.docx]

**sTable 1**. Number and age-standardized rate of incidence, prevalence, DALYs for five major musculoskeletal diseases in China, 2017

|  | Incidence, N (95% UI) | Prevalence, N (95% UI) | DALYs, N (95% UI) |
| --- | --- | --- | --- |
| **Number** | | | |
| Rheumatoid arthritis | 222,814(194,702-251,043) | 3,802,521(3,376,296-4,240,363) | 678,767(509,013-860,809) |
| Osteoarthritis | 2,745,949(2,412,717-3,127,137) | 61,190,477(54,040,132-69,208,793) | 1,971,782(983,944-3,935,656) |
| Low back pain | 27,386,551(24,018,672-30,915,260) | 63,055,701(55,414,489-70,979,527) | 7,184,540(5,081,105-9,735,270) |
| Neck pain | 18,998,802(16,656,967-21,598,036) | 87,346,162(76,052,063-98,716,541) | 8,758,013(6,066,846-12,292,429) |
| Gout | 1,644,521(1,435,340-1,886,134) | 8,331,930(7,323,633-9,412,719) | 264,729(178,292-363,196) |
| **Age standardized rate per 100,000 population** | | | |
| Rheumatoid arthritis | 12(10-13) | 196(175-218) | 35(27-45) |
| Osteoarthritis | 137(122-155) | 3,073(2,719-3,471) | 99(49-198) |
| Low back pain | 1,593(1,419-1,789) | 3,619(3,196-4,040) | 411(294-554) |
| Neck pain | 1,038(917-1,177) | 4,634(4,079-5,224) | 466(323-650) |
| Gout | 85(75-96) | 429(379-482) | 14(9-19) |

Abbreviation: UI, uncertainty interval.
