# Supplementary material for: Pattern and trend of five major musculoskeletal disorders in China from 1990 to 2017: findings from the Global Burden of Disease Study 2017
Source: BMC Med. 2021 Feb 4;19:34. doi: 10.1186/s12916-021-01905-w (PMC7860632; doi:10.1186/s12916-021-01905-w)
Supplement: Supplementary file 11 — Additional file 11: sTable 2. Population attributable fractions (PAFs) for YLDs, DALYs, and deaths related to rheumatoid arthritis, osteoarthritis, low back pain, Gout due to risk factors in China (2017). [file 12916_2021_1905_MOESM11_ESM.docx]

**sTable 2**. Population attributable fractions (PAFs) for YLDs, DALYs, and deaths related to rheumatoid arthritis, osteoarthritis, low back pain, gout due to risk factors in China (2017)

| Risk factors | Population attributable fraction | | | Age standardized population attributable fraction | | |
| --- | --- | --- | --- | --- | --- | --- |
|  | YLDs (%) | DALYs (%) | Death (%) | YLDs (%) | DALYs (%) | Death (%) |
| **Rheumatoid arthritis** |  |  |  |  |  |  |
| Smoking | 8.3(2.8,13.6) | 9.7(3.3,15.8) | 13.0(4.2,21.1) | 8.0(2.7,13.2) | 9.4(3.1,15.4) | 12.2(4.0,20.1) |
| **Osteoarthritis** |  |  |  |  |  |  |
| High body-mass index | 16.5(7.1,28.8) | 16.5(7.1,28.8) |  | 16.2(7.0,28.4) | 16.2(7.0,28.4) |  |
| **Low back pain** |  |  |  |  |  |  |
| Smoking | 16.4(12.3,20.5) | 16.4(12.3,20.5) |  | 14.6(10.9,18.4) | 14.6(10.9,18.4) |  |
| High body-mass index | 5.0(2.4,8.3) | 5.0(2.4,8.3) |  | 4.5(2.1,7.6) | 4.5(2.1,7.6) |  |
| Occupational ergonomic factors | 28.0(25.1,31.2) | 28.0(25.1,31.2) |  | 26.1(23.3,29.2) | 26.1(23.3,29.2) |  |
| **Gout** |  |  |  |  |  |  |
| High body-mass index | 24.3(11.2,41.5) | 24.3(11.2,41.5) |  | 24.0(11.1,41.1) | 24.0(11.1,41.1) |  |
| Impaired kidney function | 7.3(6.3,8.5) | 7.3(6.3,8.5) |  | 7.7(6.6,8.9) | 7.7(6.6,8.9) |  |
