# Supplementary material for: Pattern and trend of five major musculoskeletal disorders in China from 1990 to 2017: findings from the Global Burden of Disease Study 2017
Source: BMC Med. 2021 Feb 4;19:34. doi: 10.1186/s12916-021-01905-w (PMC7860632; doi:10.1186/s12916-021-01905-w)
Supplement: Supplementary file 12 — Additional file 12: sTable 3. Correlation between SDI and incidence, prevalence and DALYs. [file 12916_2021_1905_MOESM12_ESM.docx]

**sTable 3.** Correlation between SDI and incidence, prevalence and DALYs between 1990 and 2017

|  | Number | | | Age standardized rate | | |
| --- | --- | --- | --- | --- | --- | --- |
|  | Incidence | Prevalence | DALYs | Incidence | Prevalence | DALYs |
| Rheumatoid arthritis | 0.977 | 0.968 | 0.974 | 0.929 | 0.904 | 0.771 |
| Osteoarthritis | 0.984 | 0.992 | 0.992 | 0.370 | 0.640 | 0.644 |
| Low back pain | 0.934 | 0.930 | 0.932 | -0.807 | -0.808 | -0.810 |
| Neck pain | 0.998 | 0.997 | 0.997 | 0.817 | 0.754 | 0.761 |
| Gout | 0.986 | 0.985 | 0.986 | 0.953 | 0.958 | 0.965 |
