# Supplementary material for: Pattern and trend of five major musculoskeletal disorders in China from 1990 to 2017: findings from the Global Burden of Disease Study 2017
Source: BMC Med. 2021 Feb 4;19:34. doi: 10.1186/s12916-021-01905-w (PMC7860632; doi:10.1186/s12916-021-01905-w)
Supplement: Supplementary file 13 — Additional file 13: sTable 4. The value and ranking of five major musculoskeletal disorders for 19 of Group 20 countries except European Union. [file 12916_2021_1905_MOESM13_ESM.docx]

**sTable 4.** The value and ranking of five major musculoskeletal disorders for 19 of Group 20 countries except European Union

| Country | Number | | | | | | Age standardized rate | | | | | |
| --- | --- | --- | --- | --- | --- | --- | --- | --- | --- | --- | --- | --- |
|  | Incidence | rank | Prevalence | rank | DALYs | rank | Incidence | rank | Prevalence | rank | DALYs | rank |
| **Rheumatoid arthritis** | | | | | | | | | | | | |
| China | 222,814 | 2 | 3,802,521 | 2 | 678,767 | 2 | 12 | 16 | 196 | 16 | 35 | 15 |
| Indonesia | 14,601 | 11 | 225,044 | 12 | 38,909 | 12 | 6 | 19 | 91 | 19 | 16 | 19 |
| Russian Federation | 23,842 | 6 | 449,011 | 7 | 74,599 | 7 | 12 | 15 | 211 | 15 | 35 | 16 |
| Japan | 20,804 | 8 | 431,144 | 8 | 76,403 | 6 | 11 | 17 | 186 | 18 | 31 | 17 |
| South Korea | 7,931 | 14 | 147,372 | 14 | 22,611 | 14 | 11 | 18 | 187 | 17 | 29 | 18 |
| Australia | 5,089 | 19 | 91,320 | 18 | 14,963 | 18 | 16 | 6 | 268 | 8 | 43 | 9 |
| France | 15,747 | 10 | 310,709 | 9 | 46,171 | 10 | 18 | 5 | 309 | 5 | 45 | 6 |
| Germany | 26,198 | 5 | 552,051 | 5 | 78,573 | 5 | 23 | 3 | 402 | 2 | 57 | 5 |
| Italy | 12,989 | 12 | 255,849 | 11 | 40,068 | 11 | 15 | 11 | 248 | 11 | 38 | 11 |
| United Kingdom | 23,537 | 7 | 464,624 | 6 | 74,075 | 8 | 27 | 1 | 472 | 1 | 73 | 1 |
| Argentina | 7,648 | 15 | 134,965 | 15 | 22,414 | 15 | 16 | 8 | 270 | 7 | 45 | 7 |
| Canada | 7,461 | 17 | 126,741 | 16 | 21,227 | 17 | 14 | 13 | 224 | 14 | 37 | 12 |
| United States | 97,952 | 3 | 1,827,984 | 3 | 280,585 | 3 | 23 | 2 | 395 | 3 | 60 | 3 |
| Mexico | 19,825 | 9 | 307,626 | 10 | 68,401 | 9 | 16 | 9 | 253 | 10 | 57 | 4 |
| Brazil | 37,510 | 4 | 658,837 | 4 | 100,034 | 4 | 16 | 7 | 281 | 6 | 43 | 10 |
| Saudi Arabia | 5,225 | 18 | 75,931 | 19 | 10,391 | 19 | 15 | 10 | 266 | 9 | 36 | 14 |
| Turkey | 12,289 | 13 | 210,828 | 13 | 32,076 | 13 | 14 | 14 | 237 | 13 | 36 | 13 |
| India | 263,365 | 1 | 3,852,848 | 1 | 802,169 | 1 | 21 | 4 | 322 | 4 | 71 | 2 |
| South Africa | 7,620 | 16 | 118,895 | 17 | 21,615 | 16 | 14 | 12 | 240 | 12 | 44 | 8 |
| **Osteoarthritis** | | | | | | | | | | | | |
| China | 2,745,949 | 1 | 61,190,477 | 1 | 1,971,782 | 1 | 137 | 19 | 3,073 | 18 | 106 | 16 |
| Indonesia | 447,403 | 5 | 7,849,070 | 6 | 250,092 | 5 | 168 | 16 | 3,464 | 15 | 129 | 10 |
| Russian Federation | 383,696 | 7 | 8,016,726 | 5 | 249,806 | 6 | 178 | 14 | 3,495 | 14 | 145 | 5 |
| Japan | 531,220 | 4 | 14,266,652 | 4 | 450,902 | 4 | 242 | 3 | 4,720 | 3 | 159 | 2 |
| South Korea | 181,213 | 14 | 3,558,251 | 14 | 113,458 | 14 | 214 | 9 | 4,162 | 9 | 100 | 17 |
| Australia | 79,523 | 18 | 1,753,665 | 17 | 55,274 | 17 | 235 | 4 | 4,582 | 4 | 97 | 19 |
| France | 198,606 | 12 | 4,641,519 | 11 | 146,843 | 11 | 196 | 11 | 3,784 | 12 | 192 | 1 |
| Germany | 287,851 | 8 | 6,751,875 | 8 | 212,153 | 8 | 205 | 10 | 3,958 | 11 | 135 | 7 |
| Italy | 199,432 | 11 | 4,709,126 | 10 | 148,417 | 10 | 190 | 13 | 3,646 | 13 | 143 | 6 |
| United Kingdom | 216,391 | 9 | 4,867,505 | 9 | 152,894 | 9 | 218 | 8 | 4,213 | 8 | 133 | 8 |
| Argentina | 114,577 | 16 | 2,361,620 | 16 | 74,902 | 16 | 231 | 5 | 4,513 | 6 | 116 | 13 |
| Canada | 123,778 | 15 | 2,696,216 | 15 | 85,307 | 15 | 220 | 7 | 4,241 | 7 | 126 | 11 |
| United States | 1,493,494 | 3 | 32,070,714 | 3 | 1,000,398 | 3 | 317 | 1 | 6,128 | 1 | 121 | 12 |
| Mexico | 191,882 | 13 | 3,577,472 | 13 | 114,115 | 13 | 154 | 18 | 3,057 | 19 | 145 | 4 |
| Brazil | 384,383 | 6 | 7,248,484 | 7 | 230,427 | 7 | 160 | 17 | 3,143 | 17 | 133 | 9 |
| Saudi Arabia | 76,037 | 19 | 1,024,368 | 19 | 32,937 | 19 | 250 | 2 | 5,027 | 2 | 151 | 3 |
| Turkey | 209,743 | 10 | 4,060,096 | 12 | 128,630 | 12 | 228 | 6 | 4,572 | 5 | 109 | 15 |
| India | 2,461,375 | 2 | 46,548,080 | 2 | 1,466,373 | 2 | 196 | 12 | 4,114 | 10 | 109 | 14 |
| South Africa | 87,349 | 17 | 1,536,502 | 18 | 48,294 | 18 | 176 | 15 | 3,397 | 16 | 99 | 18 |
| **Low back pain** | | | | | | | | | | | | |
| China | 27,386,551 | 2 | 63,055,701 | 2 | 7,184,540 | 2 | 1,593 | 19 | 3,619 | 19 | 659 | 16 |
| Indonesia | 8,376,706 | 6 | 19,466,486 | 6 | 2,209,058 | 6 | 3,271 | 15 | 7,665 | 15 | 609 | 17 |
| Russian Federation | 7,367,971 | 7 | 17,378,612 | 7 | 1,923,931 | 7 | 3,900 | 13 | 9,025 | 14 | 1,101 | 11 |
| Japan | 8,835,324 | 5 | 22,058,738 | 5 | 2,485,176 | 5 | 5,234 | 2 | 12,615 | 2 | 1,015 | 13 |
| South Korea | 3,112,301 | 13 | 7,577,694 | 13 | 862,825 | 13 | 4,599 | 9 | 10,924 | 9 | 1,244 | 9 |
| Australia | 1,368,578 | 17 | 3,210,909 | 17 | 360,306 | 17 | 4,746 | 8 | 10,886 | 10 | 517 | 18 |
| France | 3,830,430 | 11 | 9,177,756 | 11 | 1,035,406 | 11 | 4,826 | 7 | 11,251 | 7 | 1,064 | 12 |
| Germany | 5,908,773 | 8 | 15,189,938 | 8 | 1,706,853 | 8 | 5,518 | 1 | 13,678 | 1 | 1,331 | 4 |
| Italy | 3,855,719 | 10 | 9,389,927 | 10 | 1,056,688 | 10 | 4,829 | 6 | 11,294 | 6 | 1,314 | 5 |
| United Kingdom | 4,292,106 | 9 | 10,451,180 | 9 | 1,172,394 | 9 | 5,226 | 3 | 12,362 | 3 | 1,396 | 3 |
| Argentina | 2,335,558 | 15 | 5,552,542 | 16 | 629,573 | 15 | 4,903 | 5 | 11,570 | 5 | 1,284 | 6 |
| Canada | 2,294,254 | 16 | 5,588,022 | 15 | 629,430 | 16 | 4,955 | 4 | 11,746 | 4 | 1,551 | 1 |
| United States | 16,934,266 | 3 | 39,094,815 | 3 | 4,341,290 | 3 | 4,170 | 11 | 9,515 | 12 | 1,281 | 7 |
| Mexico | 2,518,434 | 14 | 5,738,103 | 14 | 652,211 | 14 | 2,000 | 18 | 4,563 | 18 | 1,228 | 10 |
| Brazil | 10,319,872 | 4 | 25,258,207 | 4 | 2,857,277 | 4 | 4,528 | 10 | 11,018 | 8 | 1,244 | 8 |
| Saudi Arabia | 1,321,525 | 19 | 3,040,236 | 19 | 346,866 | 18 | 3,856 | 14 | 9,031 | 13 | 1,440 | 2 |
| Turkey | 3,569,388 | 12 | 8,580,790 | 12 | 967,285 | 12 | 4,086 | 12 | 9,777 | 11 | 1,005 | 14 |
| India | 31,242,531 | 1 | 70,311,160 | 1 | 7,867,245 | 1 | 2,412 | 17 | 5,478 | 17 | 862 | 15 |
| South Africa | 1,362,779 | 18 | 3,071,790 | 18 | 343,379 | 19 | 2,613 | 16 | 5,932 | 16 | 411 | 19 |
| **Neck pain** | | | | | | | | | | | | |
| China | 18,998,802 | 1 | 87,346,162 | 1 | 8,758,013 | 1 | 1,038 | 1 | 4,634 | 4 | 295 | 13 |
| Indonesia | 1,902,170 | 4 | 7,589,497 | 4 | 757,196 | 4 | 723 | 13 | 2,921 | 14 | 256 | 16 |
| Russian Federation | 1,508,431 | 5 | 6,377,916 | 6 | 620,876 | 6 | 777 | 9 | 3,177 | 11 | 442 | 5 |
| Japan | 1,490,023 | 7 | 6,855,380 | 5 | 673,702 | 5 | 752 | 10 | 3,230 | 10 | 420 | 7 |
| South Korea | 512,395 | 14 | 2,406,293 | 14 | 239,033 | 14 | 699 | 14 | 3,111 | 12 | 248 | 18 |
| Australia | 190,105 | 19 | 825,355 | 19 | 81,135 | 19 | 617 | 18 | 2,576 | 17 | 264 | 15 |
| France | 668,691 | 13 | 3,474,099 | 12 | 342,893 | 12 | 785 | 8 | 3,826 | 8 | 430 | 6 |
| Germany | 1,038,698 | 8 | 6,044,815 | 7 | 592,797 | 7 | 902 | 7 | 4,815 | 3 | 244 | 19 |
| Italy | 772,515 | 12 | 4,502,930 | 10 | 443,018 | 10 | 903 | 6 | 4,824 | 2 | 336 | 9 |
| United Kingdom | 842,409 | 10 | 4,536,811 | 9 | 445,057 | 9 | 990 | 2 | 5,005 | 1 | 496 | 1 |
| Argentina | 364,046 | 16 | 1,665,680 | 15 | 164,960 | 15 | 747 | 11 | 3,383 | 9 | 481 | 2 |
| Canada | 292,332 | 18 | 1,274,340 | 18 | 125,381 | 18 | 600 | 19 | 2,462 | 19 | 478 | 3 |
| United States | 3,890,091 | 3 | 19,446,198 | 3 | 1,890,100 | 3 | 942 | 5 | 4,385 | 6 | 382 | 8 |
| Mexico | 834,027 | 11 | 3,321,009 | 13 | 331,551 | 13 | 660 | 15 | 2,647 | 15 | 255 | 17 |
| Brazil | 1,495,206 | 6 | 5,928,272 | 8 | 588,229 | 8 | 634 | 17 | 2,506 | 18 | 310 | 12 |
| Saudi Arabia | 332,361 | 17 | 1,383,081 | 17 | 138,868 | 17 | 943 | 4 | 4,251 | 7 | 322 | 10 |
| Turkey | 863,045 | 9 | 4,015,820 | 11 | 396,934 | 11 | 966 | 3 | 4,472 | 5 | 311 | 11 |
| India | 8,433,963 | 2 | 33,097,036 | 2 | 3,263,917 | 2 | 651 | 16 | 2,612 | 16 | 289 | 14 |
| South Africa | 389,950 | 15 | 1,548,610 | 16 | 152,252 | 16 | 740 | 12 | 3,014 | 13 | 466 | 4 |
| **Gout** | | | | | | | | | | | | |
| China | 1,644,521 | 1 | 8,331,930 | 1 | 264,729 | 1 | 85 | 17 | 429 | 17 | 17 | 12 |
| Indonesia | 214,779 | 5 | 1,061,467 | 5 | 33,500 | 5 | 90 | 14 | 453 | 15 | 14 | 15 |
| Russian Federation | 191,653 | 6 | 994,793 | 7 | 30,340 | 7 | 88 | 16 | 448 | 16 | 16 | 13 |
| Japan | 290,041 | 4 | 1,933,540 | 4 | 59,974 | 4 | 118 | 5 | 735 | 6 | 18 | 11 |
| South Korea | 92,405 | 12 | 600,191 | 12 | 18,856 | 12 | 114 | 6 | 727 | 7 | 10 | 18 |
| Australia | 57,564 | 17 | 437,467 | 15 | 13,415 | 15 | 162 | 1 | 1,171 | 1 | 9 | 19 |
| France | 101,457 | 11 | 700,980 | 11 | 21,715 | 11 | 96 | 12 | 625 | 9 | 31 | 2 |
| Germany | 149,115 | 7 | 1,051,200 | 6 | 32,285 | 6 | 101 | 10 | 672 | 8 | 28 | 3 |
| Italy | 103,643 | 10 | 714,404 | 9 | 21,991 | 10 | 94 | 13 | 608 | 10 | 26 | 4 |
| United Kingdom | 114,736 | 9 | 824,209 | 8 | 25,293 | 8 | 111 | 8 | 754 | 5 | 23 | 5 |
| Argentina | 62,764 | 16 | 425,106 | 16 | 13,257 | 16 | 124 | 4 | 831 | 4 | 19 | 10 |
| Canada | 78,529 | 14 | 514,802 | 13 | 16,012 | 13 | 140 | 3 | 888 | 3 | 21 | 8 |
| United States | 689,627 | 3 | 4,839,226 | 3 | 147,693 | 3 | 146 | 2 | 996 | 2 | 20 | 9 |
| Mexico | 66,375 | 15 | 331,925 | 17 | 10,646 | 17 | 55 | 19 | 273 | 19 | 36 | 1 |
| Brazil | 141,630 | 8 | 701,857 | 10 | 22,230 | 9 | 61 | 18 | 301 | 18 | 23 | 7 |
| Saudi Arabia | 27,555 | 19 | 138,013 | 19 | 4,399 | 19 | 111 | 7 | 592 | 11 | 23 | 6 |
| Turkey | 85,918 | 13 | 450,325 | 14 | 14,037 | 14 | 97 | 11 | 508 | 13 | 14 | 16 |
| India | 1,051,296 | 2 | 5,182,936 | 2 | 160,684 | 2 | 90 | 15 | 453 | 14 | 14 | 14 |
| South Africa | 50,658 | 18 | 264,289 | 18 | 8,167 | 18 | 105 | 9 | 563 | 12 | 14 | 17 |
